# AKR1C3 Converts Castrate and Post-Abiraterone DHEA-S into Testosterone to Stimulate Growth of Prostate Cancer Cells via 5-Androstene-3β,17β-Diol

**DOI:** 10.1158/2767-9764.CRC-23-0235

**Published:** 2023-09-19

**Authors:** Andrea J. Detlefsen, Clementina A. Mesaros, Ling Duan, Trevor M. Penning

**Affiliations:** 1 Department of Biochemistry and Biophysics, University of Pennsylvania, Philadelphia, Pennsylvania.; 2 Department of Systems Pharmacology and Translational Therapeutics, University of Pennsylvania, Philadelphia, Pennsylvania.; 3 Center of Excellence in Environmental Toxicology, Perelman School of Medicine, University of Pennsylvania, Philadelphia, Pennsylvania.

## Abstract

Androgen receptor signaling inhibitors (ARSI) are used to treat castration-resistant prostate cancer (CRPC) to stop a resurgence of androgen receptor (AR) signaling. Despite early success, patients on ARSIs eventually relapse, develop drug resistance, and succumb to the disease. Resistance may occur through intratumoral steroidogenesis mediated by upregulation of aldo-keto reductase family 1C member 3 (AKR1C3). Patients treated with leuprolide (castrate) and those treated with leuprolide plus abiraterone (post-Abi) harbor a reservoir of DHEA-S which could fuel testosterone (T) biosynthesis via AKR1C3 to cause a resurgence of prostate cancer cell growth. We demonstrate that concentrations of DHEA-S found in castrate and post-Abi patients are (i) converted to T in an AKR1C3-dependent manner in prostate cancer cells, and (ii) in amounts sufficient to stimulate AKR1C3-dependent cell growth. We observed this in primary and metastatic prostate cancer cell lines, CWR22PC and DuCaP, respectively. Androgen measurements were made by stable isotope dilution LC-MS/MS. We demonstrate AKR1C3 dependence using stable short hairpin RNA knockdown and pharmacologic inhibitors. We also demonstrate that free DHEA is reduced to 5-androstene-3β,17β-diol (5-Adiol) by AKR1C3 and that this is a major metabolite, suggesting that in our cell lines 5-Adiol is a predominant precursor of T. We have identified a mechanism of ARSI resistance common to both primary and metastatic cell lines that is dependent on the conversion of DHEA to 5-Adiol on route to T catalyzed by AKR1C3.

**Significance:**

We show that reservoirs of DHEA-S that remain after ARSI treatment are converted into T in primary and metastatic prostate cancer cells in amounts sufficient to stimulate cell growth. Pharmacologic and genetic approaches demonstrate that AKR1C3 is required for these effects. Furthermore, the route to T proceeds through 5-Adiol. We propose that this is a mechanism of ARSI drug resistance.

## Introduction

Prostate cancer is the most common cancer among men in the United States (1). Prostate cancer is driven by androgen receptor (AR) activation, where testosterone (T) and 5α-dihydrotestosterone (DHT) are the most potent AR ligands and are responsible for the majority of downstream AR signaling (2). As a result, prostate cancer therapy aims to reduce circulating androgens to castrate levels (<20 ng/dL; ref. 3). This therapeutic goal can be achieved by surgical or chemical castration (e.g., leuprolide) which will prevent T production in the Leydig cells of the testes (4). While the initial response to treatment is good, patients can develop resistance and progress to the lethal form of the disease termed castration-resistant prostate cancer (CRPC; refs. 5, 6). CRPC is characterized by a resurgence of AR signaling that fuels growth of existing tumors or appearance of new metastases despite castrate levels of serum T/DHT (6). Treatment for CRPC aims to inhibit AR signaling with the use of therapeutics known as AR signaling inhibitors (ARSI), e.g., Abiraterone (Abi) and Enzalutamide (Enz; refs. 7–12). Abi inhibits CYP17A1 and prevents adrenal DHEA biosynthesis, while Enz acts as an AR antagonist (8, 10–12). Despite the early success of ARSIs, a subset of patients develops resistance and succumb to the disease.

**FIGURE 1:**
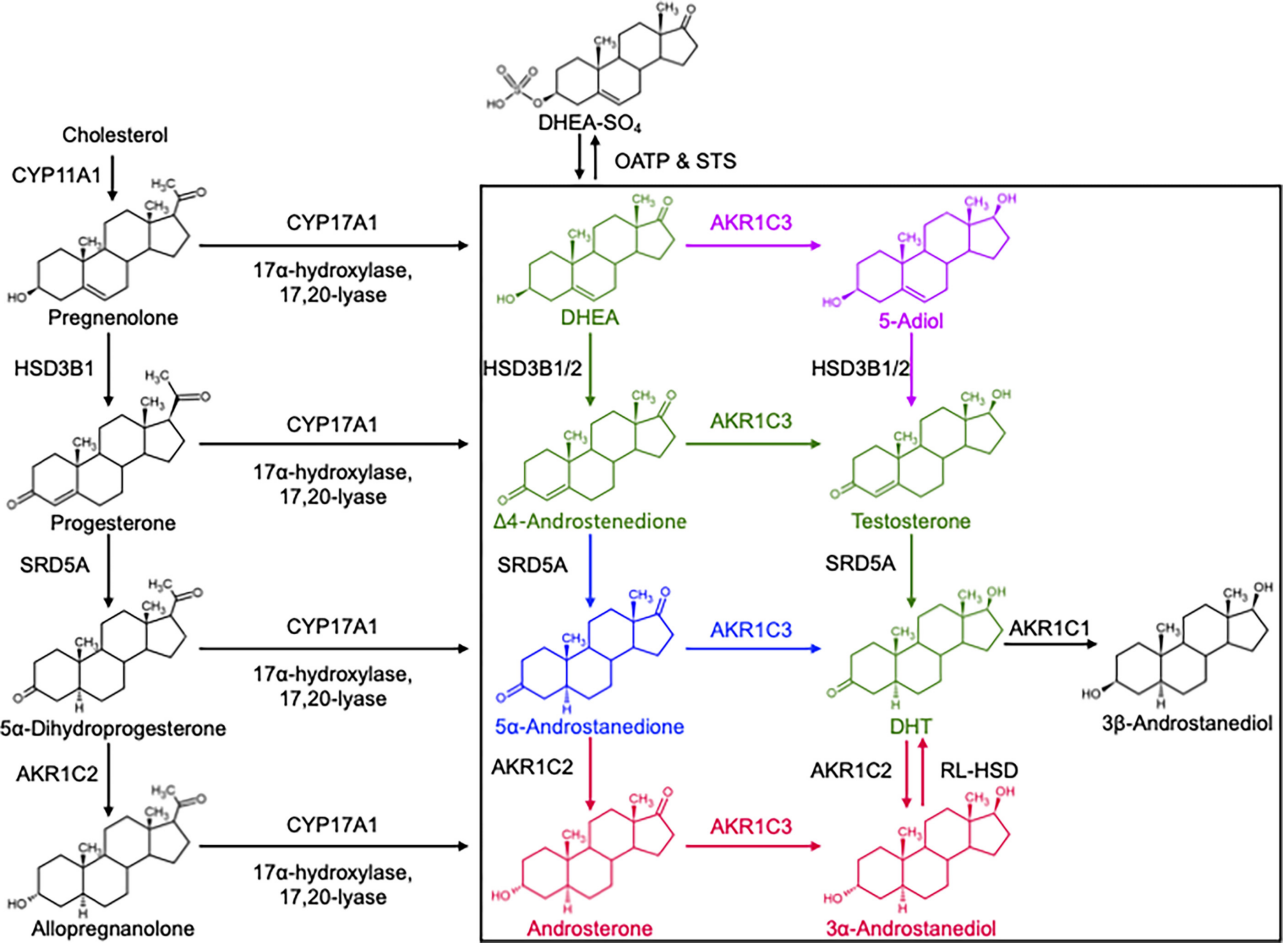
Androgen biosynthetic pathways. Black rectangle indicates intraprostatic reactions. Classical pathway in green, alternative in blue, backdoor in red, and 5-Adiol in purple.

ARSI drug resistance can arise through two general mechanisms. One originates from changes in the AR, such as gene amplification, mutations, splice variants, and changes in coactivators and corepressors (13–17). The other resistance mechanism occurs through intratumoral steroidogenesis (18). Here, circulating androgen precursors from the adrenal gland are converted within prostate cancer cells to potent androgens by steroidogenic enzymes that are upregulated in this disease state (19). Different combinations of precursor androgen and steroidogenic enzymes can give rise to diverse resistance pathways. In the network of reactions that generate T and DHT within prostate cells, there exist four possible androgen biosynthesis pathways (Fig. 1). The canonical, backdoor, and alternative pathways have been identified to participate in prostate cancer androgen biosynthesis using different experimental models, conditions, and modes of detection (Fig. 1; refs. 20–26). It is evident from these studies that the supplemented precursor determines the entry point into the metabolic network, which in addition to the expression levels of several competing and interconnected steroidogenic enzymes, influences which pathway is dominant. In patients that develop resistance, treatment history and stage of the disease, tumor heterogeneity, and even differences between metastatic sites in the same person could all impact the major source of precursor and the resulting biosynthetic pathway. However, a common element of these pathways is the expression of type 5 17β-hydroxysteroid dehydrogenase also known as aldo-keto reductase family 1C member 3 (AKR1C3). AKR1C3 catalyzes the 17-ketosteroid reduction of Δ4-androstenedione (4AD) to T, 5α-Androstanedione (5α-AD) to DHT, and Androsterone (AD) to 3α-Androstanediol (3α-Adiol; Fig. 1; refs. 27, 23). It is inferred that it also reduces DHEA to 5-Adiol, an immediate precursor of T, but this has not been previously demonstrated. AKR1C3 is upregulated in CRPC and overexpressed in cell culture in response to androgen deprivation and in prostate cancer cells made resistant to Abi and Enz (19, 28–30). Recent work has also expanded the field's understanding of the role of AKR1C3 by demonstrating its ability to act as an AR coactivator and to stabilize both wild-type (WT) AR and the AR-V7 splice variant (23, 31, 32).

To investigate the efficacy of androgen ablation, we collaborated on an intense androgen deprivation therapy clinical trial in which patients with localized high-risk prostate cancer received leuprolide plus Abi acetate and prednisone (33). Serum androgen measurements were made: before treatment (baseline), after 12 weeks of leuprolide (castrate), and after an additional 12 weeks of leuprolide plus Abi and prednisone (post-Abi). Baseline DHEA-SO_4_ (DHEA-S) was measured at 5 µmol/L, whereas serum T was 15 nmol/L (34). Castrate levels of DHEA-S after leuprolide remained at 5 µmol/L while serum T dropped to 0.17 nmol/L (34). Furthermore, the castrate level of DHEA-S achieved would likely exist in patients post-Enz treatment, which as an AR antagonist would not affect DHEA-S levels (35–37). Post-Abi DHEA-S was measured at 0.5 µmol/L, whereas T dropped slightly further to 0.05 nmol/L (34). Notably, both the castrate and post-Abi levels of DHEA-S dwarf the baseline levels of circulating T (15 nmol/L). This led us to hypothesize that the reservoir of DHEA-S that remains after castration and post-Abi is sufficient to produce T In amounts that will stimulate prostate cancer growth and that this conversion would be AKR1C3 dependent. Furthermore, this could be a general mechanism of ARSI resistance. The study described here aimed to test this hypothesis in both primary and metastatic prostate cancer cell lines.

## Materials and Methods

### Cell Culture

CWR22PC and DuCaP WT cells were grown in maintenance media containing RPMI1640 (Gibco #11875-085) with 10% FBS (Cytiva #SH30071.03), 2 mmol/L l-Glutamine (Gibco #25030-081), and 1% Pen Strep (Gibco #15140-122). CWR22PC AKR1C3 knockdown (KD) and DuCaP AKR1C3 KD cells were maintained in selection media containing RPMI1640 with 10% FBS, 2 mmol/L l-Glutamine, 1% Pen Strep, and 1 µg/mL puromycin (Gibco #A1113802). CWR22PC and DuCaP metabolism experiments were performed in treatment media containing phenol red-free RPMI (Gibco #11835030) with 5% charcoal dextran stripped FBS (Sigma #F6765), 1% Pen Strep, and 2 mmol/L l-Glutamine. The CWR22PC cells (RRID CVCL_LI38) were obtained in 2013. DuCaP cells (RRID CVCL_2025) were obtained from ATCC after deposition by the laboratory of Dr. Ken Pienta at Johns Hopkins School of Medicine in 2013. CWR22PC and DuCaP cell lines were authenticated by short-terminal repeat assays that indicate the allele detected at each locus tested, where each allele represents the number of short tandem repeats present at that locus. The resulting DNA profile uniquely identifies an individual cell line. Cell lines were determined to be *Mycoplasma* free using a DNA-based PCR test that can detect 19 species of *Mycoplasma* that include the most commonly encountered *Mycoplasma* species that contaminate cell culture. Cells were thawed and cultured over a period of 2 months for experiments before thawing a fresh vial.

### Generating AKR1C3 Short Hairpin RNA Stable KD Cell Lines

A human lentiviral vector with a microRNA-adapted short hairpin RNA (shRNA) sequence for AKR1C3 knockdown (target gene sequence 5′-GGAGTAAATTGCTAGATTT-3′) and a TurboGFP fluorescent protein-target reporter were purchased from Dharmacon Reagents (Perkin Elmer). Cells were seeded at 1 × 10^4^ CWR22PC cells or 2 × 10^5^ DuCaP cells per well in a 12-well plate in maintenance media. A total of 24 hours later, media was replaced with 0.4 mL of serum-free media with a multiplicity of infection of 10. Six hours after transduction, an additional 1 mL of maintenance media was added to each well. Cells were incubated overnight. Forty-eight hours after transduction, cells were examined under a microscope for the expression of the reporter gene (TurboGFP) as a first indication of transduction efficiency. Seventy-two hours after transduction, maintenance media was replaced with selection media containing 1 µg/mL puromycin. Approximately 20 days later (dependent on cell growth), single colonies were picked and passaged into a 6-well plate with puromycin selection media. Individual colonies were expanded into three 60 mm dishes and assayed for AKR1C3 expression by qRT-PCR and Western blot (WB) analysis.

### qRT-PCR

RNA was extracted from a 10 cm plate with 350 µL lysis buffer with 2-mercaptoethanol according to RNeasy minikit (Qiagen #74104) and treated with RNase-free DNase (Qiagen #79254). RNA was then reverse transcribed to cDNA using a high-capacity RNA to cDNA kit (Applied Biosystems #4387406). qRT-PCR reactions were conducted on a MJ Research PTC-2000 Peltier Thermal Cycler. The conditions used to measure AKR1C3 mRNA expression by qRT-PCR were 95°C for 15 minutes followed by 40 cycles of: 94°C for 15 seconds, then 57°C for 30 seconds, then 72°C for 30 seconds. The conditions used to measure HSD17B3, RODH4, and RL-HSD mRNA expression by qRT-PCR were 95°C for 15 minutes followed by 40 cycles of: 94°C for 30 seconds, then 60°C for 30 seconds, then 72°C for 30 seconds. The conditions used to measure GAPDH mRNA expression by qRT-PCR were 95°C for 15 minutes followed by 40 cycles of: 94°C for 30 seconds, then 60°C for 30 seconds, then 72°C for 30 seconds. AKR1C3 mRNA was measured with the forward primer 5′-GAAGTAAAGCTTTGGAGGTC-3′ and reverse primer 5′-GTCAACATAGTCCAATTGAGC-3′. HSD17B3 mRNA was measured with the forward primer 5′-TTGGAGGTGAAACCTGTGGCTG-3′ and reverse primer 5′-CTACCTGACCTTGGTGTTGAGC-3′. RODH4 mRNA was measured with the forward primer 5′-TATGGCGTGGAAGCCTTCTCTG-3′ and reverse primer 5′-GGTCCCAAATCTCCAGGAAGCT-3′. RL-HSD mRNA was measured with the forward primer 5′-CCAGCATTCTGGGAAGAGTTGC-3′ and reverse primer 5′-CCGTTCTGAAGTAGCCAGGTTC-3′. GAPDH mRNA was measured with the forward primer 5′-CATCTCTGCCCCCTCTGCTGA-3′ and reverse primer 5′-GGATGACCTTGCCCACAGCCT-3′. Expression of each gene was calculated as a ratio of fg to fg of GAPDH in reference to a standard curve for each gene. Standard curves for each gene of interest were generated using full-length standards (2,500,000 to 0.025 fg) as previously described by us (38). Full-length standards for AKR1C3, HSD17B3, RODH4, and RL-HSD were generated by restriction digests of plasmids pcDNA3-AKR1C3, pcDNA3-RODH4, pcDNA3-RLHSD, and pcDNA3-HSD17B3 (a gift from Professor Jerzy Adamski). A full-length standard for GAPDH was generated by PCR amplification of GAPDH template cDNA using forward primer 5′-TCCTCCTGTTCGACAGTCAG**-**3′ and reverse primer 5′-CACAGACACCCCATCCT-3′ data were analyzed using GraphPad Prism 9.

### WB Analysis

Cell extracts were harvested from a 10 cm plate with 250 µL of RIPA buffer (Pierce #89900) with 1:100 protease inhibitor cocktail (Sigma #P-8340). Samples were sonicated using a QSonica Q500 and then centrifuged for 10 minutes at 15,000 RPM at 4°C. Protein extracts (40 µg) were heated for 10 minutes at 100°C in Laemmli buffer and run on a 4%–15% SDS polyacrylamide gel (Bio-Rad #4561086). Gels were transferred to nitrocellulose membrane and blocked with 5% blocking buffer (Bio-Rad #1706404) in TBS containing 0.1% tween-20 (TBST) for 1 hour at room temperature. Blots were then incubated with primary antibody 1:500 murine anti-human AKR1C3 (39), or 1:3,000 murine anti-human α-Tubulin (Novus Biologicals #NB100-690) in 5% blocking buffer TBST overnight at 4°C. AKR1C3 antibody was validated to be monospecific against a panel of recombinant human AKRs. It was also monospecific against AKR1C3 stably transfected LNCaP cells and in transiently transfected HEK-293 cells, demonstrating no cross-reactivity with other proteins in cell lysates. Blots were then incubated with secondary antibody 1:2,000 murine-IgG BP-HRP (Santa Cruz Biotechnology #sc-525409) in 5% blocking buffer in TBST for 1 hour at room temperature. Blots were then developed using Pierce enhanced chemiluminescence Western blotting substrate (Thermo Fisher Scientific #32106) and imaged using a Bio-Rad ChemiDoc MP imaging system.

### Cell Treatments and Sample Processing for Stable Isotope Dilution LC-MS/MS

Cells were seeded in 2 mL of treatment media into 6-well plates with 2 × 10^6^ cells per well. A total of 24 hours after seeding cells, media was replaced with treatment media (phenol red-free, CD-FBS) containing either 0.5 or 5 µmol/L DHEA-S (Steraloids #A8334-000) ± 30 µmol/L ASP-9521 (synthesized by the Winkler group at the University of Pennsylvania, Philadelphia, PA) or BMT4-159 (40). The mode of action, pharmacologic details, and structures are provided for each inhibitor in Supplementary Table S1. Synthetic compounds were characterized by ^1^H-NMR and HRMS, and purity was found to be greater than 95% by reversed-phase high-performance liquid chromatography (RP-HPLC) with UV detection. For metabolic pathway experiments, cells were treated with 5 µmol/L DHEA-S. Directly before cell media was harvested at the indicated timepoints, 500 pg of each internal standard was spiked into each well 2,3,4-[^13^C_3_]-T, 2,3,4-[^13^C_3_]-DHT, and 2,3,4-[^13^C_3_]-DHEA (Cambridge Isotope Laboratories #CLM-9164-PK, #DLM-3023-PK, #CLM-10784-PK). Androgens were then isolated by liquid-liquid extraction with 2 mL of diethyl ether twice. The upper organic layer containing androgens and internal standards were combined and dried down by vacuum centrifugation. The extracted androgens were then derivatized with picolinic acid (PA) as described below.

### PA Derivatization

The extracted androgens were derivatized with PA to form picolinate esters on the hydroxy groups to increase ionization and detection sensitivity by mass spectrometry (Supplementary Fig. S1A). A derivatization mixture was made directly before use by dissolving 20 mg 4-(dimethylamino)pyridine (Sigma-Aldrich #107700), 50 mg 2-PA (Sigma-Aldrich #P42800), and 40 mg 2-methyl-6-nitrobenzoic anydride (TCI #M1439) in 1 mL of anhydrous acetic acid (Acros Organics #326811000). A total of 100 µL of the derivatization mixture was added to each tube of dried androgens, followed by 40 µL of triethylamine (Sigma #90340). Androgens were derivatized for 90 minutes while shaking at 500 rpm. Reactions were quenched with 1 mL of 10% acetic acid in water, followed by a solid-phase extraction (SPE) cleanup. C-18 SPE columns (Phenomenex #8B-S001-DAM) were set up on a vacuum manifold system and preconditioned with 1 mL methanol followed by 1 mL of water. Quenched samples were then flowed through the column to waste, then columns were washed three times with 1 mL of water followed by three times with 1 mL 30% acetonitrile acid in water. Androgens were eluted with 2 mL of acetonitrile. Samples were then dried down by vacuum centrifugation and resuspended in 100 µL of 60% acetonitrile in water.

### Stable Isotope Dilution LC-MS/MS

A Vanquish UPLC system (Thermo Fisher Scientific) was coupled to a TSQ Atlis mass spectrometer (Thermo Fisher Scientific) for androgen quantitation. T, Epitestosterone (Epi-T), DHEA, AD, DHT, Epiandrosterone (Epi-AD), 5-Adiol, 3α-Androstanediol (3α-Adiol), and 3β-Androstanediol (3β-AdioL) were purchased from Steraloids. Derivatized androgens were chromatographically separated using a Kinetex C18 (Phenomenex #O00D-4462-AN) 100 mm × 2.1 mm, 2.6 µmol/L 100 Å column with a C18 guard column 2.1 mm internal diameter. Solvent A was 0.05% (v/v) formic acid in water, solvent B was 0.05% formic acid in 4:6 acetonitrile:methanol, and the flow rate was 0.25 mL/minute. The method began at 20% B for 1 minute, then increased to 60% B over 5 minutes and maintained at 60% B for 20 minutes. B then increased to 95% over 15 minutes and was maintained at 95% for 5 minutes. B was then decreased to 20% over 5 minutes and maintained at 20% B for 15 minutes. The mass spectrometer operating conditions were 4,000 V spray voltage, 350°C ion transfer capillary temperature, 1.5 mTor collision gas (argon), and positive ion polarity. Xcalibur 2.6 software (Thermo Fisher Scientific) was used for data acquisition and processing. Androgens in samples were quantified in reference to calibration curves for each androgen that were constructed from the peak area ratio of each standard to corresponding internal standard plotted against the total pg of the androgen in the vial with 1/x weighting in Xcalibur QuanBrowser (Supplementary Fig. S1B and S2). The statistical significance of androgen measurements was evaluated by one-way ANOVA using GraphPad Prism.

### Growth Curve

Cells were seeded in 100 µL of treatment media (phenol red-free, CD-FBS) into 96-well plates. CWR22PC cells were seeded at a density of 10,000 cells per well, and DuCaP cells were seeded at a density of 20,000 cells per well. Note, cells were seeded 48 hours before the start of the experiment to allow ample time for cells to attach to the wells. On day 1, 100 µL of treatment media containing 10 or 1 µmol/L DHEA-S ± 60 µmol/L ASP-9521 or BMT4-159 was added to each well for a final treatment concentration of 5 or 0.5 µmol/L DHEA-S ± 30 µmol/L ASP-9521 or BMT4-159. Cells were then imaged and counted using a BioTek Cytation 5 plate reader for the next 9 days. Cell growth was reported as a percentage of the cell count on day 1. The statistical significance of differences in cell growth on day 10 was evaluated by one-way ANOVA using GraphPad Prism.

### RP-HPLC Kinetics

The NADPH-dependent reduction of DHEA (5 µg of protein, 0.27 µmol/L per reaction) was monitored in the previously determined linear range of the velocity versus protein concentration curve. DHEA reduction was measured in 1 mL systems which contained 0–30 µmol/L DHEA, 180 µmol/L NADPH, 4% acetonitrile, 0.1 mol/L potassium phosphate (pH 6.0) and were run for 1 hour at 37°C. All samples were extracted twice with 2 mL ethyl acetate and dried by vacuum centrifugation before derivatization with PA as described above. Samples were then reconstituted in 100 µL of 60% acetonitrile in water and were run on a Zorbax ODS 5 µm 4.6 × 250 mm column (Waters #880952-702) using a constant flow rate of 0.5 mL/minute with solvent A of 0.05% (v/v) formic acid in water, and solvent B of 0.05% formic acid in 4:6 acetonitrile:methanol, for quantification of substrate and product in the reactions via reversed-phase high-performance liquid chromatography linked to UV detection (RP-UV/HPLC). The method began at 20% B for 1 minute, then increased to 60% B over 5 minutes and maintained at 60% B for 20 minutes. B then increased to 95% over 15 minutes and was maintained at 95% for 5 minutes. B was then decreased to 20% over 5 minutes and maintained at 20% B for 15 minutes. Elution of DHEA and 5-Adiol was monitored by the chromophore of the picolinate group at 225 and 264 nm. Peaks were integrated and products were quantified from calibration curves constructed using derivatized authentic standards. Waters Alliance 2695 Separations Module and Waters 996 Photodiode Array Detector were used for all RP-UV/HPLC measurements. All reactions were performed in triplicate and Michaelis–Menten kinetic parameters were obtained using GraphPad Prism.

### Data Availability Statement

Data were generated by the authors and available on request.

## Results

### AKR1C3 shRNA Stable KD Cell Lines (qRT-PCR and WB of AKR1C3)

**FIGURE 2:**
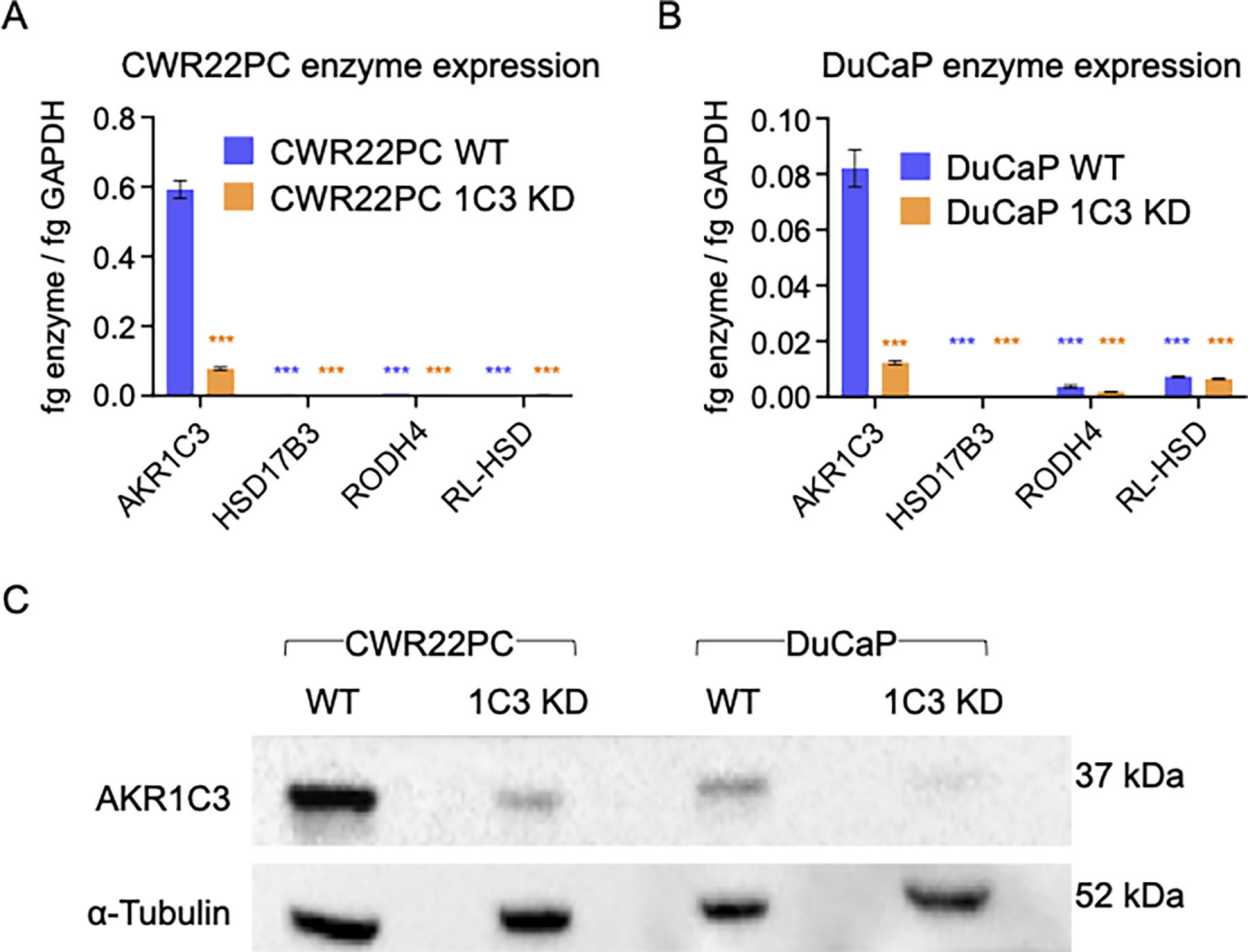
AKR1C3 expression in WT versus KD cell lines. qRT-PCR shows fg transcript/fg GAPDH for CWR22PC WT and AKR1C3 KD cells (**A**), and DuCaP WT and AKR1C3 KD cells (**B**). AKR1C3 protein expression for CWR22PC WT versus AKR1C3 KD and DuCaP WT versus AKR1C3 KD with α-Tubulin loading control (**C**). *P* values represented by *** is *P* < 0.0001. AKR1C3 expression is compared between WT and AKR1C3 KD cell lines using one-way ANOVA. Expression of other 17-oxidoreductase enzymes are compared with AKR1C3 expression within respective cell lines (WT or 1C3 KD) using a one-way ANOVA.

To assess the AKR1C3 mRNA expression in CWR22PC KD and DuCaP KD cells relative to WT cells, qRT-PCR was performed in Fig. 2A and B. AKR1C3 protein expression in WT and AKR1C3 KD cell lines was probed by WB analysis and is shown in Fig. 2C. Notably, CWR22PC WT cells expressed more AKR1C3 than DuCaP WT cells (Fig. 2). CWR22PC and DuCaP cell lines were also screened for expression of three additional 17β-hydroxysteroid dehydrogenases: HSD17B3, the major 17-ketosteroid reductase in Leydig cells (41), and RODH4 and RL-HSD, which can exhibit 3-ketosteroid oxidase activity (42). The mRNA expression of HSD17B3, RODH4, and RL-HSD was negligible in both cell lines compared with AKR1C3 mRNA expression (Fig. 2A and B).

### T Formation in CWR22PC and DuCaP Cells

**FIGURE 3:**
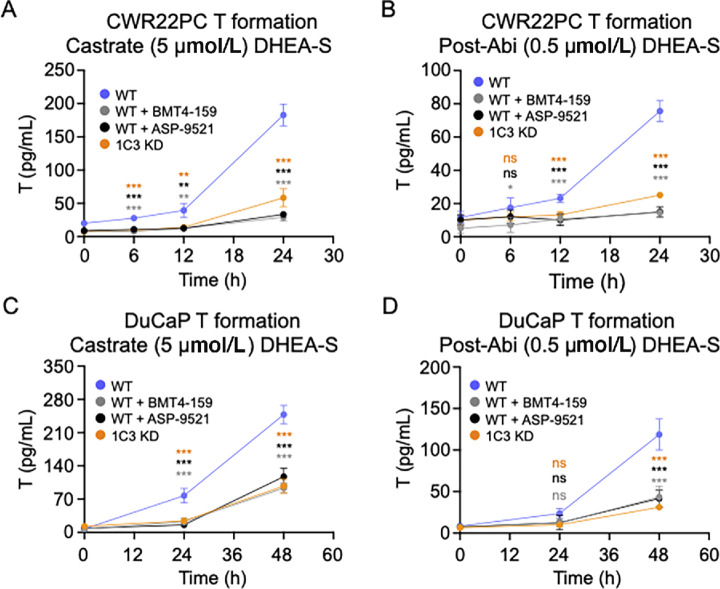
DHEA-S to T metabolism measured by SID-LC-MS/MS. T formation from castrate (5 µmol/L) DHEA-S (**A**) and post-Abi (0.5 µmol/L) DHEA-S (**B**) in CWR22PC WT cells ± ASP-9521 or BMT4-159 and in CWR22PC AKR1C3 KD cells. T formation from castrate (5 µmol/L) DHEA-S (**C**) and post-Abi (0.5 µmol/L) DHEA-S (**D**) in DuCaP WT cells ± ASP-9521 or BMT4-159 and in DuCaP AKR1C3 KD cells. *P* values indicated where ***, *P* < 0.0001; **, *P* < 0.001; and *, *P* < 0.01 as compared with WT DHEA-S metabolism at all timepoints. Statistical significance was analyzed by a one-way ANOVA.

CWR22PC and DuCaP cells formed T from both the castrate (5 µmol/L) and post-Abi (0.5 µmol/L) treatment levels of DHEA-S in an AKR1C3-dependent manner, where KD or inhibition of AKR1C3 attenuated T production. CWR22PC WT cells treated with 5 µmol/L DHEA-S formed 182 ± 16 pg T/mL at the 24-hour timepoint, whereas CWR22PC 1C3 KD cells formed only 58 ± 13 pg T/mL by the same timepoint (Fig. 3A). Similarly, T production in CWR22PC WT cells treated with 5 µmol/L DHEA-S plus 30 µmol/L ASP-9521 or BMT4-159 was reduced to 33 ± 3 and 28 ± 6 pg T/mL at 24 hours, respectively (Fig. 3A). CWR22PC WT cells treated with 0.5 µmol/L DHEA-S formed 75 ± 6 pg T/mL at the 24-hour timepoint, whereas CWR22PC 1C3 KD cells formed only 25 ± 1 pg T/mL by the same timepoint (Fig. 3B). Similarly, T production in CWR22PC WT cells treated with 0.5 µmol/L DHEA-S plus 30 µmol/L ASP-9521 or BMT4-159 was reduced to 15 ± 3 and 16 ± 2 pg T/mL at 24 hours, respectively (Fig. 3B). CWR22PC 1C3 KD cells produced slightly more T than WT cells treated with an AKR1C3 inhibitor. This could reflect the presence of the small amount of AKR1C3 remaining in the KD cells, as the knockdown of AKR1C3 expression was not 100% complete. The addition of AKR1C3 inhibitors to CWR22PC 1C3 KD cells eliminated any residual T production showing it to be entirely AKR1C3 dependent (Supplementary Fig. S3A and S3B).

DuCaP WT cells treated with 5 µmol/L DHEA-S formed 248 ± 19 pg T/mL at the 48-hour timepoint, whereas DuCaP 1C3 KD cells formed only 97 ± 15 pg T/mL by the same timepoint (Fig. 3C). Similarly, T production in DuCaP WT cells treated with 5 µmol/L DHEA-S plus 30 µmol/L ASP-9521 or BMT4-159 was reduced to 117 ± 17 and 77 ± 17 pg T/mL at 24 hours, respectively (Fig. 3C). DuCaP WT cells treated with 0.5 µmol/L DHEA-S formed 118 ± 18 pg T/mL at the 48-hour timepoint, whereas DuCaP 1C3 KD cells formed only 31 ± 2 pg T/mL by the same timepoint (Fig. 3D). Similarly, T production in DuCaP WT cells treated with 0.5 µmol/L DHEA-S plus 30 µmol/L ASP-9521 or BMT4-159 was reduced to 43 ± 12 and 22 ± 3 pg T/mL at 24 hours, respectively (Fig. 3D). The addition of AKR1C3 inhibitors to DuCaP 1C3 KD cells treated with 5 µmol/L DHEA-S eliminated any residual T production (Supplementary Fig. S3C and S3D). Notably, DuCaP T formation was monitored over a 48-hour time course, whereas CWR22PC cells were monitored over 24 hours. CWR22PC cells were found to produce more T over a shorter period than DuCaP cells. This is likely because CWR22PC WT cells express higher levels of AKR1C3 than DuCaP WT cells (Fig. 2).

### Cell Growth in CWR22PC and DuCaP Cells

**FIGURE 4:**
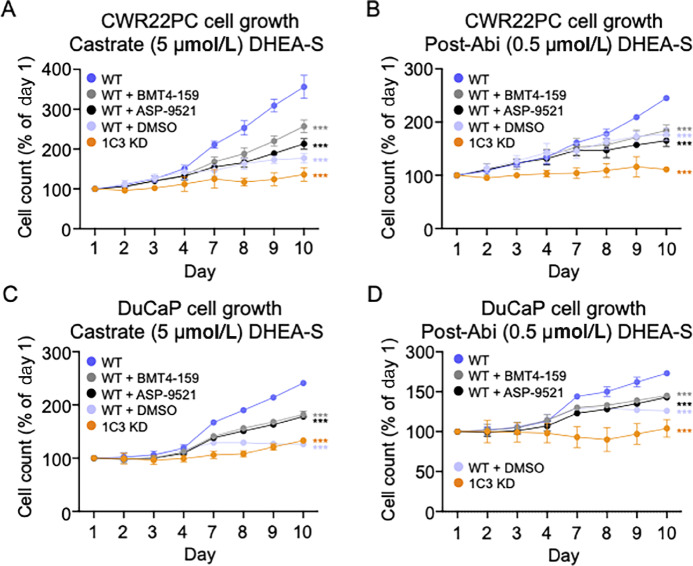
DHEA-S stimulated cell growth. Cell growth from castrate (5 µmol/L) DHEA-S (**A**) and post-Abi (0.5 µmol/L) DHEA-S (**B**) in CWR22PC WT cells ± ASP-9521 or BMT4-159 and in CWR22PC AKR1C3 KD cells. Cell growth from castrate (5 µmol/L) DHEA-S (**C**) and post-Abi (0.5 µmol/L) DHEA-S (**D**) in DuCaP WT cells ± ASP-9521 or BMT4-159 and in DuCaP AKR1C3 KD cells. *P* values indicated where ***, *P* < 0.0001; **, *P* < 0.001; and *, *P* < 0.01 compared with growth of WT cells treated with DHEA-S at the 10-day timepoint. Statistical significance was determined by a one-way ANOVA.

CWR22PC and DuCaP cell growth was induced by both the castrate (5 µmol/L) and post-Abi (0.5 µmol/L) treatment levels of DHEA-S in an AKR1C3-dependent manner, where KD or inhibition of AKR1C3 reduced cell growth. CWR22PC WT cell growth is attenuated in the absence of androgens (DMSO control), where cells only exhibited 177 ± 12% growth at day 10. Addition of either 5 or 0.5 µmol/L DHEA-S at induced cell growth to 356 ± 29% and 245 ± 5% growth by day 10, respectively (Fig. 4A and B). However, CWR22PC 1C3 KD cell growth was not induced by either concentration of DHEA-S (Fig. 4A and B). The addition of either 30 µmol/L ASP-9521 or BMT4-159 to CWR22PC WT cells treated with 5 µmol/L DHEA-S reduced cell growth to 213 ± 13% and 257 ± 16% at day 10, respectively. Similarly, AKR1C3 inhibitors reduced growth of CWR22PC WT cells treated with 0.5 µmol/L DHEA-S to 165 ± 11% and 184 ± 11% by day 10, respectively. Growth of CWR22PC 1C3 KD cells treated with AKR1C3 inhibitors can be found in Supplementary Fig. S4A and S4B.

Similarly, DuCaP WT cell growth is attenuated in the absence of androgens, where cells only exhibit 126 ± 1% growth at day 10. Addition of either 5 or 0.5 µmol/L DHEA-S induced cell growth to 241 ± 2 and 173 ± 1% growth by day 10, respectively (Fig. 4C and D). However, DuCaP 1C3 KD cell growth was not induced by either concentration of DHEA-S (Fig. 4C and D). The addition of either 30 µmol/L ASP-9521 or BMT4-159 to DuCaP WT cells treated with 5 µmol/L DHEA-S reduced growth to 178 ± 3 and 182 ± 6% at day 10, respectively. Similarly, AKR1C3 inhibitors reduced growth of DuCaP WT cells treated with 0.5 µmol/L DHEA-S to 143 ± 1% and 145 ± 1% by day 10, respectively. Growth curves of DuCaP 1C3 KD cells treated with AKR1C3 inhibitors can be found in Supplementary Fig. S4C and S4D.

### DHEA to 5-Adiol Kinetic Parameters

**FIGURE 5:**
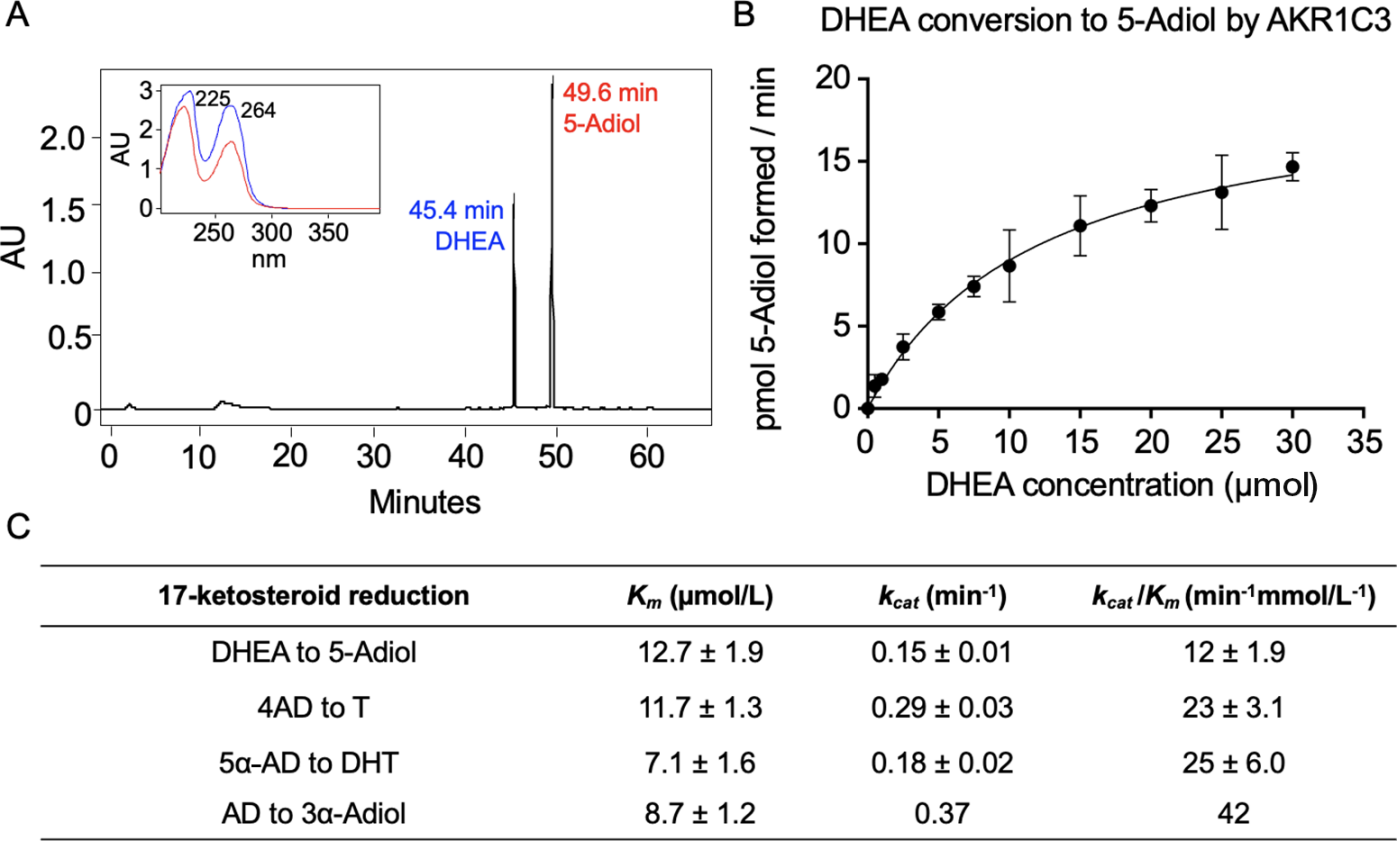
AKR1C3 conversion of DHEA to 5-Adiol kinetics determined by RP-HPLC. RP-HPLC separation of PA derivatized DHEA and 5-Adiol (**A**), V versus S curve for AKR1C3 conversion of DHEA to 5-Adiol (**B**), comparison of kinetic parameters of AKR1C3 DHEA to 5-Adiol reduction (RP-HPLC, pH 6.0), 4AD to T (RP-HPLC, pH 6.0), 5α-AD to DHT (RP-HPLC, pH 7.0), and AD to 3α-Adiol (spectrophotometric, pH 7.0; **C**).

AKR1C3 has 17-ketosteroid reductase activity capable of reducing DHEA to 5-Adiol, 4AD to T, 5α-AD to DHT, and Androsterone to 3α-Androstanediol (Fig. 1; ref. 27). Each of these reactions is a part of the four routes for biosynthesis of T and DHT in prostate cancer cells. The conversion of 4AD to T is characteristic of the canonical pathway, the conversion 5α-AD to DHT is characteristic of the alternate pathway, the conversion of androsterone to 3α-androstanediol is characteristic of the backdoor pathway, and the conversion of DHEA to 5-Adiol represents the 5-Adiol pathway (27). Therefore, AKR1C3 is implicated in all pathways. While the kinetic parameters for the former three reactions have been previously determined (Fig. 5A; refs. 25, 43), those for the conversion of DHEA to 5-Adiol have not. To assess whether conversion of DHEA to 5-Adiol was comparable with the other 17-ketosteroid reductions catalyzed by AKR1C3, we determined kinetic parameters for this reaction. First, an RP-HPLC method was developed to separate PA derivatized DHEA and 5-Adiol in which the latter is detected as a bis-picolinate (Fig. 5B). Then, a velocity versus (substrate) curve was generated using the discontinuous RP-HPLC assay (Fig. 5C). AKR1C3 conversion of DHEA to 5-Adiol was found to have a *K_m_* of 12.7 ± 1.9 µmol/L, *k_cat_* of 0.15 ± 0.01 minute^−1^, and *k_cat_*/*K_m_* of 12 ± 1.9 minute^−1^ mmol/L^−1^. These kinetic parameters were similar to those reported for the conversion of 4AD to T measured by discontinuous RP-HPLC assay which gave a *K_m_* of 11.7 ± 1.3, *k_cat_* of 0.29 ± 0.03 minute^−1^ and *k_cat_*/*K_m_* of 23 ± 3.1 minute^−1^ mmol/L^−1^ at pH 6.0 where steroid derivtization was unnecessary (44). The conversion of 5α-AD to DHT was reported to have a *K_m_* of 7.1 ± 1.6 µmol/L, *k_cat_* of 0.18 ± 0.02 minute^−1^, and *k_cat_*/*K_m_* of 25 ± 6.0 minute^−1^ mmol/L^−1^ using dinitrophenylhydrazine to derivatize these transparent steroids by RP-HPLC (43). The reduction of AD to 3α-Adiol measured spectrophotometrically was reported to have *K_m_* of 8.7 ± 1.2 µmol/L, *k_cat_* of 0.37 minute^−1^, and *k_cat_*/*K_m_* of 42 minute^−1^ mmol/L^−1^ (25). Catalytic efficiencies for these four reactions fall within a 2-fold range of each other. From these results, we conclude that the conversion of DHEA to 5-Adiol is a viable transformation catalyzed by AKR1C3 that could likely occur in prostate cancer cells at the same level as other pathways.

### Metabolic Pathway DHEA-S to T

**FIGURE 6:**
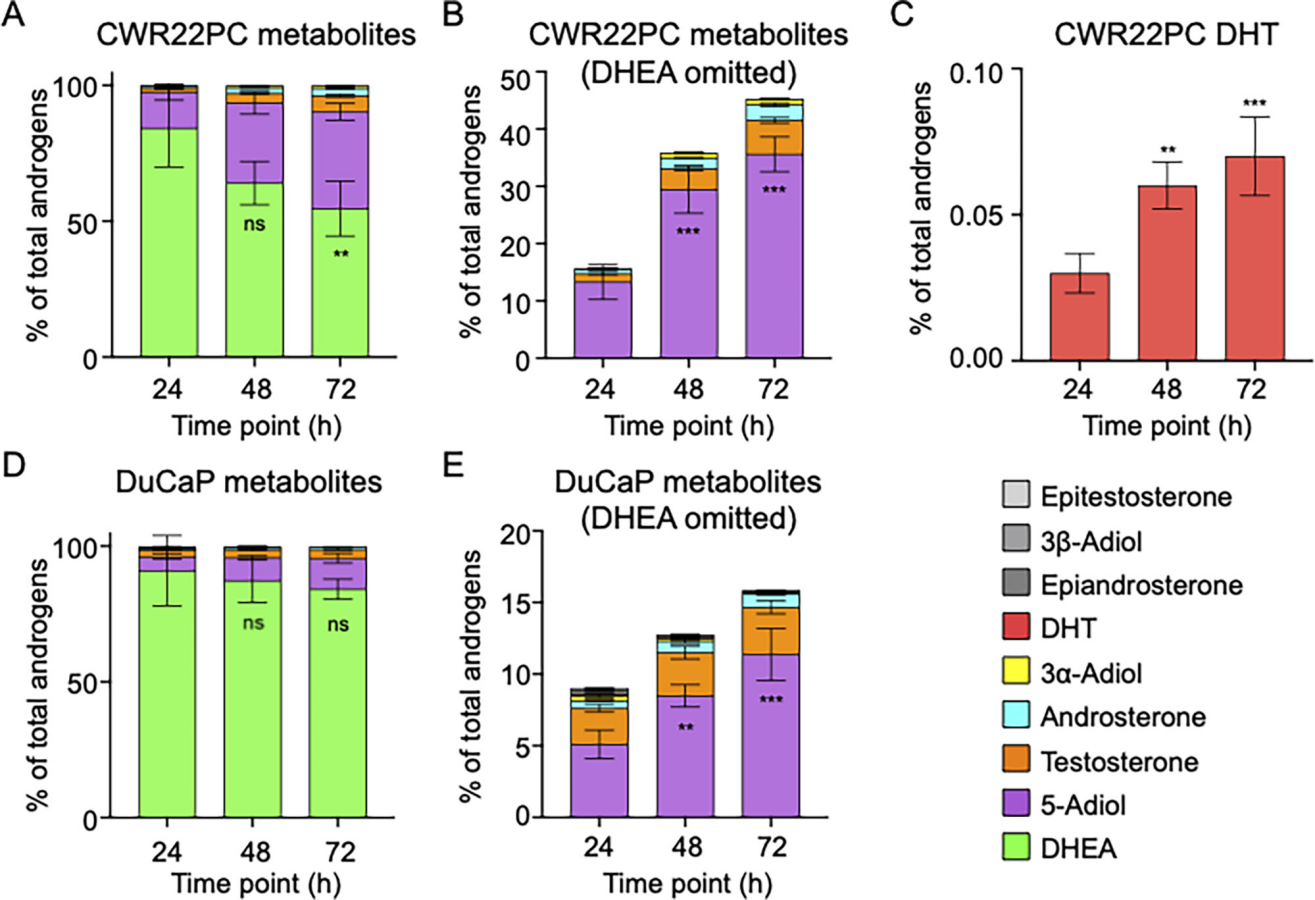
DHEA-S to T metabolic pathway analysis by SID-LC-MS/MS. Percentage of total of each androgen measured from 5 µmol/L DHEA-S treatment at each timepoint in CWR22PC WT cells (**A**), percentage of total of each androgen measured downstream of DHEA from 5 µmol/L DHEA-S treatment at each timepoint in CWR22PC WT cells (**B**), percentage of total DHT measured in CWR22PC WT cells from 5 µmol/L DHEA-S treatment (**C**), percentage of total of each androgen measured from 5 µmol/L DHEA-S treatment at each timepoint in DuCaP WT cells (**D**), percentage of total of each androgen measured downstream of DHEA from 5 µmol/L DHEA-S treatment at each timepoint in DuCaP WT cells (**E**). *P* values indicated where ***, *P* < 0.0001 and **, *P* < 0.001 as compared with 24 hours timepoint for each metabolite. A analyzes DHEA, B analyzes 5-Adiol, C analyzes DHT, D analyzes DHEA, and E analyzes 5-Adiol.

To investigate metabolism routes of DHEA-S in our cell lines, we expanded the panel of androgens detected by stable isotope dilution LC-MS/MS (SID-LC-MS/MS) to measure the hydroxy-androgens DHEA, 5-Adiol, T, Epitestosterone, DHT, 3α-Androstanediol, 3β-Androstanediol, Androsterone, and Epiandrosterone. Over 72 hours, DHEA makes up the majority of the total amount of androgens measured in CWR22PC WT cells at each timepoint. However, it decreased over time from 84.2 ± 14.3%, to 64.1 ± 7.9%, to 54 ± 10.1% of the total at 24, 48, and 72 hours, respectively (Fig. 6A). This could be due to rapid transport and sulfatase cleavage of DHEA-S, resulting in a quick initial accumulation of DHEA that then is further metabolized to other androgens. The next most abundant androgen was 5-Adiol (Fig. 6A). At 24 hours, 5-Adiol made up 13.3 ± 3.0% of all measured androgens and increased to 29.5 ± 4.2% at 48 hours, and 35.6 ± 3.1% at 72 hours. Closer examination of the non-DHEA metabolites in Fig. 6B additionally shows formation of T, androsterone, and 3α-Adiol, in decreasing order. Similar to 5-Adiol, these androgens increasd in percentage over time, reflecting continuing metabolism of DHEA-S over 72 hours. Further analysis in Fig. 6C reveals a small amount of DHT formation in CWR22PC WT cells; however, it is comparatively dwarfed by T production.

Similarly, in the DuCaP WT cells over a 72-hour time course, DHEA made up the majority of the total amount of androgens measured at each timepoint (Fig. 6D). At 24 hours, DHEA made up 90.0 ± 12.9% of measured androgens which decreased to 87.3 ± 7.9% at 48 hours, and to 84.2 ± 3.7% at 72 hours. The next most abundant measured androgen was 5-Adiol (Fig. 6D). At 24 hours, 5-Adiol made up 5.1 ± 1.0% of measured androgens which increased to 8.5 ± 0.8% at 48 hours, and 11.4 ± 1.8% at 72 hours. A closer look at the non-DHEA metabolites in Fig. 6E shows formation of T, Androsterone, and 3α-Adiol, in decreasing order. Similar to 5-Adiol, these androgens increased in percentage over time, reflecting continued downstream metabolism of DHEA-S over 72 hours.

Notably, the percentage of androgens downstream from DHEA (5-Adiol, T, Androsterone, 3α-Androstanediol, DHT, Epiandrosterone, 3β-Andostanediol, and Epitestosterone) was lower in DuCaP cells. Furthermore, unlike in CWR22PC cells, DHT production was not detected in DuCaP cells. This is consistent with the slower rate of T formation and growth previously observed in DuCaP cells compared with CWR22PC (Fig. 3A vs. C and Fig. 4A vs. C). These observations are likely due to higher AKR1C3 protein expression in CWR22PC WT versus DuCaP WT (Fig. 2). Overall, the large formation of 5-Adiol compared with other intermediates suggests that the 5-Adiol pathway is utilized in DHEA-S metabolism by both cell lines.

## Discussion

Herein, we identified a potential mechanism of ARSI drug resistance that involves the conversion of castrate and post-Abi levels of DHEA-S to T in a manner that is AKR1C3 dependent. We monitored T production and cell growth in both primary and metastatic prostate cancer cell lines after delivering the amounts of DHEA-S found previously in the serum of patients at two different stages of prostate cancer treatment: castrate and post-Abi. We confirmed that the level of DHEA-S that prevails after either treatment is metabolized to T and drives prostate cancer cell growth. Furthermore, these affects were AKR1C3 dependent, as T/DHT production and cell growth were eliminated by either AKR1C3 knockdown or small-molecule inhibitors of AKR1C3. It is likely that the castrate level of DHEA-S that exists after 12 weeks of leuprolide would be similar to the level of DHEA-S circulating in patients post-Enz, which as an AR antagonist is not expected to affect DHEA-S levels (35–37). We predict that DHEA-S could also serve as a reservoir of intratumoral steroidogenesis post-Enz treatment. Enz is five to eight times more potent than the older generation AR antagonist, *R*-bicalutamide, for the AR (10). However, AR affinity for endogenous ligands still exceeds that of Enz and intratumoral T/DHT formation could outcompete Enz (45, 46).

AKR1C3 upregulation is a hallmark of CRPC, and has been replicated in cell culture models of androgen deprivation where AKR1C3 overexpression confers resistance to Abi and Enz (19, 28–30). Despite AKR1C3’s impact in prostate cancer steroidogenesis, it is potential as a treatment in the clinic remains untapped. The singular completed first phase I/Ib trial tested the Astellas AKR1C3 inhibitor ASP-9521 and reported that while it was well tolerated, it was not effective (47). However, a major flaw in this study design was the exclusion of patients who had received androgen deprivation therapy through Abi or *R*-bicalutamide (47). The authors comment that the lack of efficacy of ASP-9521 could be due to low AKR1C3 expression in participants (47). We agree with this point and believe that the excluded group of patients that had received prior treatment with ARSI would likely have the highest levels of AKR1C3 expression and be most likely to benefit from an AKR1C3 inhibitor. Another ongoing phase I/II clinical trial aims to test the efficacy of the AKR1C3 inhibitor indomethacin in patients with CRPC that progress after Enz and will notably include measurement of AKR1C3 levels (48). We are hopeful that this preselection of patients that have advanced disease and have progressed on Abi will benefit from AKR1C3 inhibitors.

The canonical (20, 21), alternate (22, 23), and backdoor pathways (24–26) have all been claimed as major androgen biosynthetic pathways downstream of various androgen precursors. However, the 5-Adiol pathway has not been well studied. The RP-HPLC method used here allowed us for the first time to measure the ability of recombinant AKR1C3 to reduce free DHEA to 5-Adiol. We found that this steroid conversion had very similar catalytic efficiencies to those reported for other 17-ketosteroid substrates. The sensitivity of our SID-LC-MS/MS method also allowed us to interrogate DHEA-S metabolism in cells. We found that there was low overall conversion of DHEA-S to T in both CWR22PC and DuCaP cell lines which could reflect low efficiency of steps upstream of androgen formation such as transport into cells by organic anion transporters and/or cleavage of the DHEA-S sulfate group by steroid sulfatases. Although the amount of DHEA-S converted to T was less than 0.01% of the total added, this was sufficient to cause prostate cancer cell growth. We also found that a major intermediate steroid metabolite that accumulated was 5-Adiol, suggesting that the pathway for DHEA metabolism to T proceeds through 5-Adiol. The slow conversion of 5-Adiol to T is indicative of the expected low activity of HSD3B1 which is considered a rate determining step in prostate cancer androgen metabolism which would also be consistent with its saturation kinetics displayed at high DHEA concentrations (49, 50). This slow conversion could suggest that 5-Adiol might accumulate without being converted to T. However, accumulation of 5-Adiol would likely cause growth attenuation because 5-Adiol is a substrate for estrogen receptor β which slows prostate cancer cell growth and growth attenuation is not observed (51, 52). In these same cells, the amount of T produced from DHEA-S was much greater than DHT. Notably, aside from the canonical pathway, DHEA-S metabolism through 5-Adiol results in the least number of enzymatic steps to from T compared with the alternate and backdoor pathway. While others have suggested that a pathway that by-passes T formation may be favored (22), it is likely that the pathway observed depends on the precursor used. We believe that DHEA-S is the most relevant precursor for intratumoral androgen biosynthesis in the castrate male.

We found slight differences in the effects of AKR1C3 inhibitors versus AKR1C3 knockdown by shRNA when measuring T production and cell growth induced by DHEA-S. Knockdown of AKR1C3 had a more pronounced effect than inhibitors on cell growth in the CWR22Pc cells. Conversely, inhibitors more strongly reduced T production. This observed difference between the efficacy of AKR1C3 pharmacologic and genetic knockdown could stem from AKR1C3’s nonenzymatic functions. Monofunctional competitive AKR1C3 inhibitors block the enzymatic function of AKR1C3, but leave AKR1C3 coactivator function intact that could still support some AR signaling and lead to cell growth. However, knockdown of AKR1C3 would block both enzymatic and nonenzymatic functions and would appear more effective in the growth assays.

Our data support the concept that AKR1C3 is a preeminent target in CRPC whose overexpression can exacerbate the disease via its steroidogenic activity. AKR1C3 conversion of DHEA-S remaining in patients after leuprolide, Abi, and potentially Enz therapy may feed intratumoral steroidogenesis to replenish AR ligands within prostate cancer cells and contribute to ARSI drug resistance.

## Supplementary Material

Supplemental Table 1 Structures of AKR1C3 Inhibitors

Supplemental Figure 1 Supplemental Figure 1 shows the picolinic acid derivatization scheme and SIC-LC-MSMS chromatographic separation of picolinic acid derivatized hydroxyandrogens.

Supplemental Figure 2 Supplemental Figure 2 shows standard curves generated for hydroxyandrogens using SIC-LC-MS/MS.

Supplemental Figure 3 Supplemental Figure 3 shows DHEA-S to T metabolism in AKR1C3 KD cell lines as measured by SID-LC-MS/MS.

Supplemental Figure 4 Supplemental Figure 4 shows DHEA-S stimulated cell growth in AKR1C3 KD cell lines.
