# Supplementary material for: AKR1C3 Converts Castrate and Post-Abiraterone DHEA-S into Testosterone to Stimulate Growth of Prostate Cancer Cells via 5-Androstene-3β,17β-Diol
Source: Cancer Res Commun. 2023 Sep 19;3(9):1888–98. doi: 10.1158/2767-9764.CRC-23-0235 (PMC10508215; doi:10.1158/2767-9764.CRC-23-0235)
Supplement: Supplemental Table 1 — Structures of AKR1C3 Inhibitors [file crc-23-0235-s01.pdf]

Supplemental Table 1. Structures of AKR1C3 Inhibitors

Inhibitor	Mode of Action	IC_{50} Value	Structure
ASP-9521	Competitive Inhibitor	290 nM*	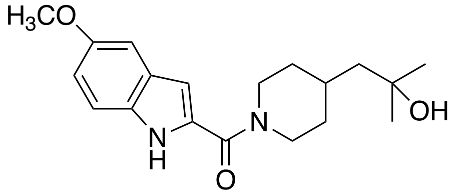
BMT4-159	Competitive Inhibitor	60 nM**	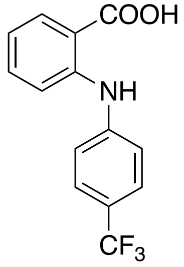

*Reduction of 4-androstene-3,17-dione to testosterone (Wangtrakuldee et al. (2019) *J Steroid Biochem. Mol. Biol.* 192, 105283).

**Oxidation of *S*-tetralol to tetralone (Adeniji et al. (2011) *Bioorg. Med. Lett.* 21, 1464)
