# Supplementary material for: AKR1C3 Converts Castrate and Post-Abiraterone DHEA-S into Testosterone to Stimulate Growth of Prostate Cancer Cells via 5-Androstene-3β,17β-Diol
Source: Cancer Res Commun. 2023 Sep 19;3(9):1888–98. doi: 10.1158/2767-9764.CRC-23-0235 (PMC10508215; doi:10.1158/2767-9764.CRC-23-0235)
Supplement: Supplemental Figure 1 — shows the picolinic acid derivatization scheme and SIC-LC-MSMS chromatographic separation of picolinic acid derivatized hydroxyandrogens. [file crc-23-0235-s02.pdf]

Supplemental Figure 1

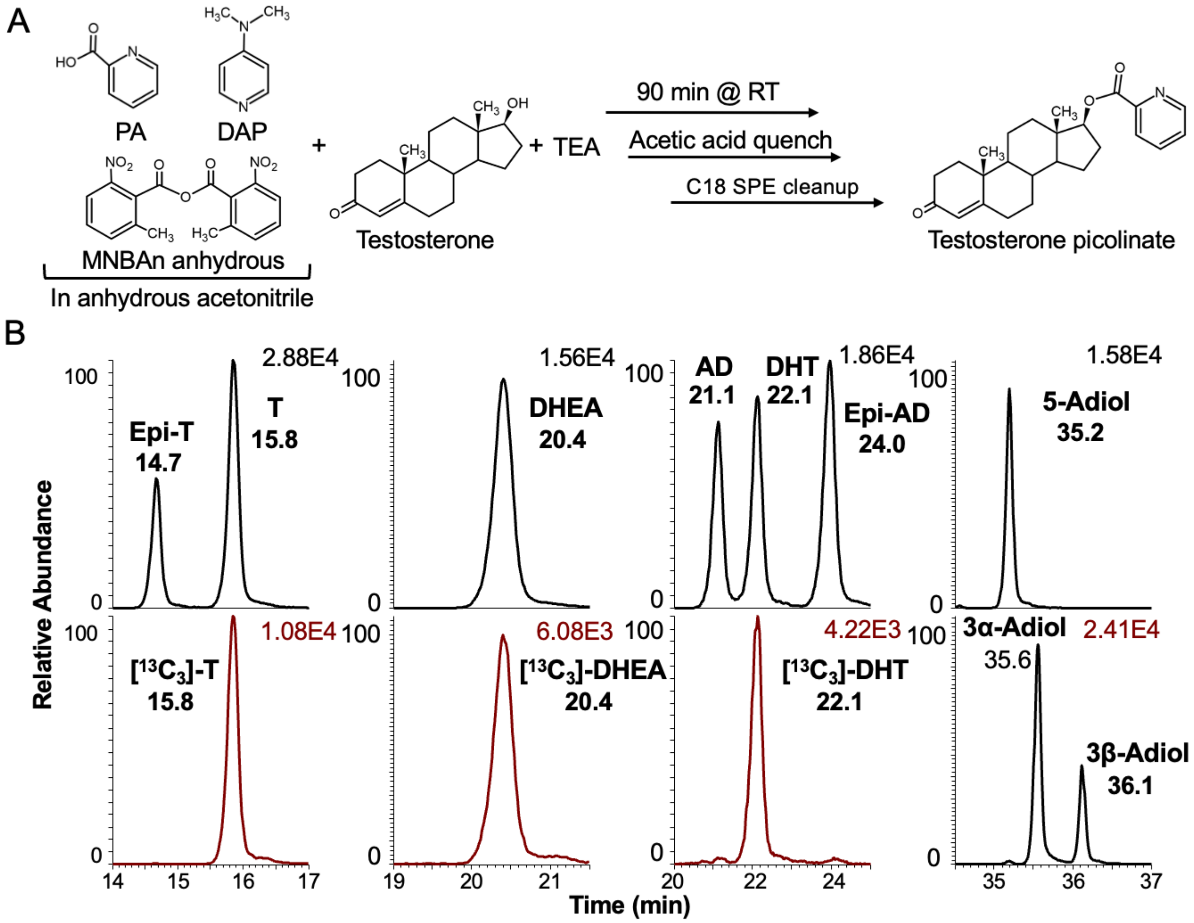

Supplemental Fig 1 *Picolinic acid derivatization and SID-LC-MS/MS chromatographic separation. a) picolinic acid derivatization reaction scheme, b) SID-LC-MS/MS ion chromatogram showing separation of hydroxy-androgen and internal standards.*
