# Supplementary material for: AKR1C3 Converts Castrate and Post-Abiraterone DHEA-S into Testosterone to Stimulate Growth of Prostate Cancer Cells via 5-Androstene-3β,17β-Diol
Source: Cancer Res Commun. 2023 Sep 19;3(9):1888–98. doi: 10.1158/2767-9764.CRC-23-0235 (PMC10508215; doi:10.1158/2767-9764.CRC-23-0235)
Supplement: Supplemental Figure 2 — shows standard curves generated for hydroxyandrogens using SIC-LC-MS/MS. [file crc-23-0235-s03.pdf]

Supplemental Figure 2

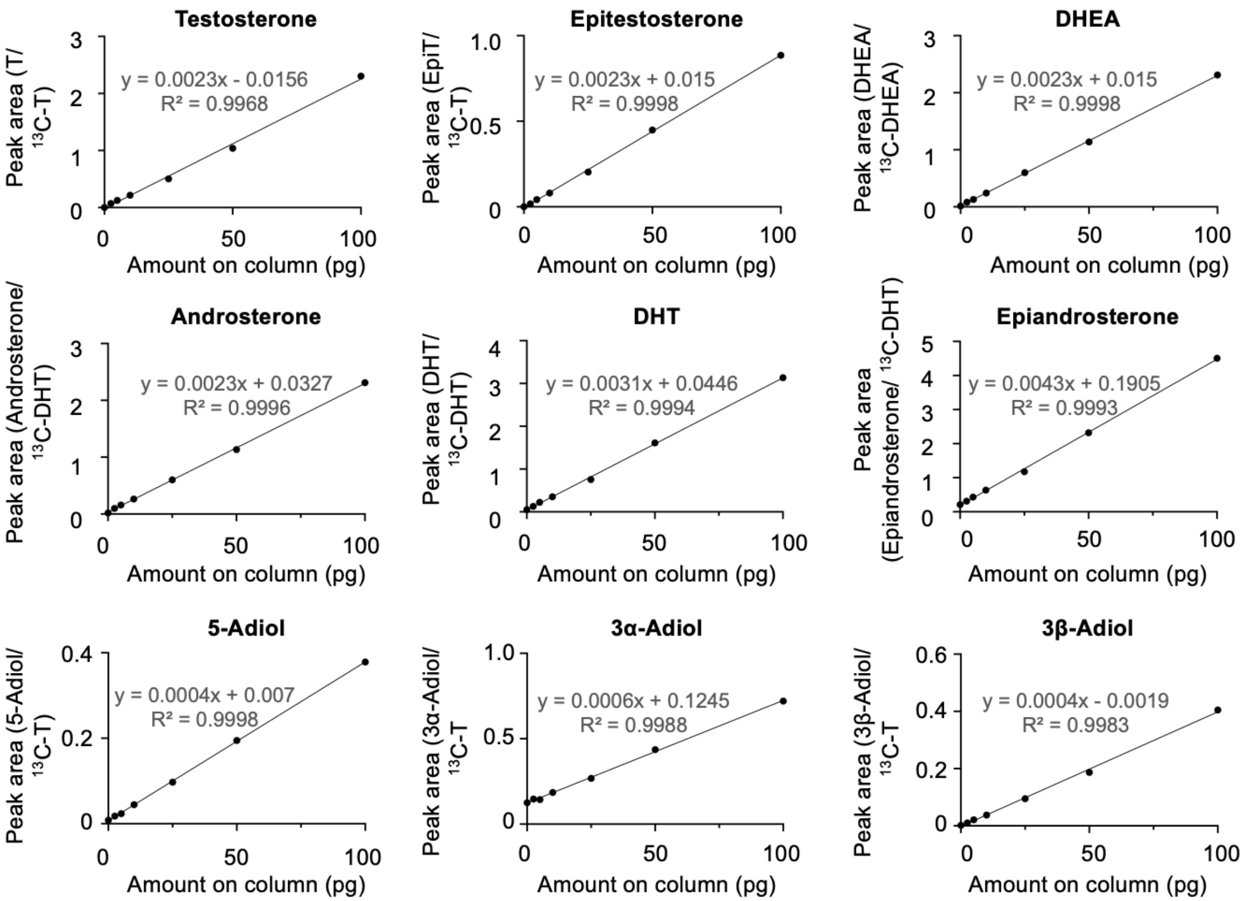

Supplemental Figure 2 *Standard curves generated for each hydroxy-androgen measured by SID-LC-MS/MS.*
