# Supplementary material for: AKR1C3 Converts Castrate and Post-Abiraterone DHEA-S into Testosterone to Stimulate Growth of Prostate Cancer Cells via 5-Androstene-3β,17β-Diol
Source: Cancer Res Commun. 2023 Sep 19;3(9):1888–98. doi: 10.1158/2767-9764.CRC-23-0235 (PMC10508215; doi:10.1158/2767-9764.CRC-23-0235)
Supplement: Supplemental Figure 3 — shows DHEA-S to T metabolism in AKR1C3 KD cell lines as measured by SID-LC-MS/MS. [file crc-23-0235-s04.pdf]

Supplemental Figure 3

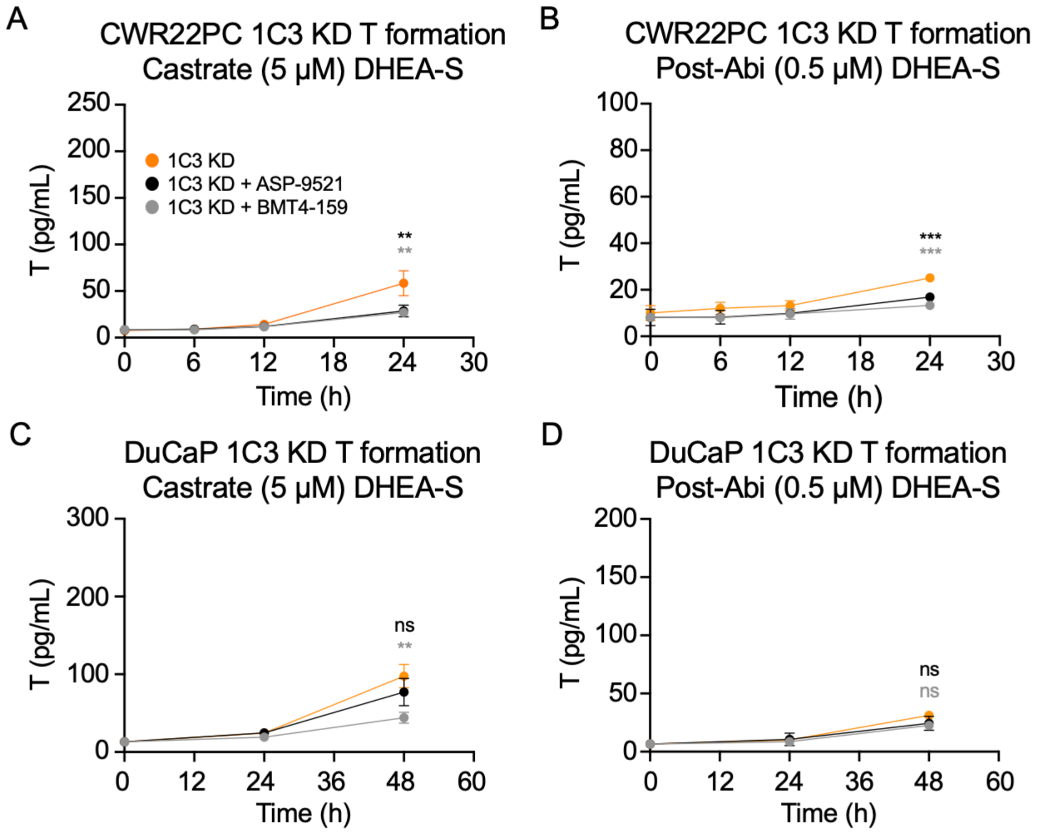

Supplemental Figure 3 *DHEA-S to T metabolism in AKR1C3 KD cells as measured by SID-LC-MS/MS. T formation from a) castrate (5 μM) DHEA-S and b) post-Abi (0.5 μM) DHEA-S in CWR22PC AKR1C3 KD cells +/- ASP-9521 or BMT4-159. T formation from c) castrate (5 μM) DHEA-S and d) post-Abi (0.5 μM) DHEA-S in DuCaP AKR1C3 KD cells +/- ASP-9521 or BMT4-159. P values indicated where *** is p<0.0001, and ** is p<0.001 as compared to AKR1C3 KD cell DHEA-S metabolism at either 24 h (CW22Pc KD cells) or 48 h (DuCaP KD cells)*
