# Supplementary material for: AKR1C3 Converts Castrate and Post-Abiraterone DHEA-S into Testosterone to Stimulate Growth of Prostate Cancer Cells via 5-Androstene-3β,17β-Diol
Source: Cancer Res Commun. 2023 Sep 19;3(9):1888–98. doi: 10.1158/2767-9764.CRC-23-0235 (PMC10508215; doi:10.1158/2767-9764.CRC-23-0235)
Supplement: Supplemental Figure 4 — shows DHEA-S stimulated cell growth in AKR1C3 KD cell lines. [file crc-23-0235-s05.pdf]

Supplemental Figure 4

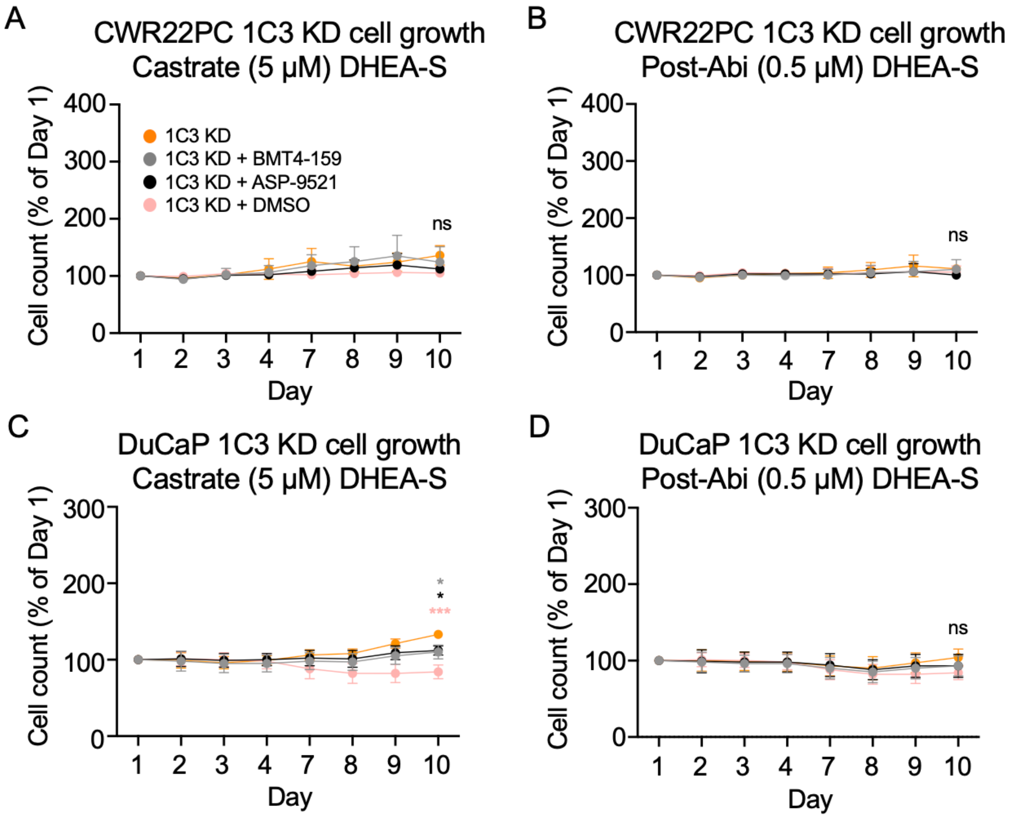

Supplemental Figure 4 *DHEA-S stimulated cell growth in AKR1C3 KD cells. Cell growth from a) castrate (5 µM) DHEA-S and b) post-Abi (0.5 µM) DHEA-S in CWR22PC AKR1C3 KD cells +/- ASP-9521 or BMT4-159. Cell growth from c) castrate (5 µM) DHEA-S and d) post-Abi (0.5 µM) DHEA-S in DuCaP WT cells +/- ASP-9521 or BMT4-159. P values indicated where *** is p<0.0001 and * is p<0.01 compared to growth of AKR1C3 KD cells treated with DHEA-S at 10 days.*
